# The Relationship Between Empowering Motivational Climate in Physical Education and Social Adaptation of Senior High School Students: An Analysis of Chain Mediating Effect

**DOI:** 10.3389/fpsyg.2022.854279

**Published:** 2022-05-17

**Authors:** Kelei Guo, Qishuai Ma, Shujun Yao, Chao Liu, Zhen Hui, HuaSheng Chen, Pengfei Wen

**Affiliations:** ^1^School of Physical Education and Health, Zhaoqing University, Zhaoqing, China; ^2^School of Physical Education, Huaibei Normal University, Huaibei, China; ^3^School of Marxism, Zhaoqing University, Zhaoqing, China; ^4^Guangzhou Sontan Polytechnic College, Guangzhou, China; ^5^School of Physical Education, Guangzhou Sport University, Guangzhou, China

**Keywords:** empowering motivational climate in physical education, social adaptation, physical education engagement, emotional intelligence, senior high school students

## Abstract

This study aims to contribute to understanding the mechanisms underlying the association between empowering motivational climate in physical education and social adaptation among senior high school students, and has important implications for interventions that aim at improving social adaptation among senior high school students. Through the quota sampling, 1,526 students (average age = 17 years, SD = 0.714 years) who came from Anhui Province and met the requirements participated and completed the Empowering Motivational Climate Questionnaire in Physical Education (EMCQ-PE), the Physical Education Engagement Scale (PEES-S), the Emotional Intelligence Scale (EIS) (Chinese version), and the Adolescent Social Adaptation Assessment Questionnaire (ASAAQ). For data analysis, Pearson’s correlation analysis, structural equation model test, and bias-corrected percentile Bootstrap method were carried out in turn. The results showed that empowering motivational climate in physical education positively predicted social adaptation (β = 0.282, *p* < 0.01), empowering motivational climate in physical education positively predicted physical education engagement and emotional intelligence (β = 0.169, *p* < 0.01; β = 0.690, *p* < 0.01), physical education engagement positively predicted emotional intelligence and social adaptation (β = 0.591, *p* < 0.01; β = 0.058, *p* < 0.05), and emotional intelligence positively predicted social adaptation (β = 0.365, *p* < 0.01). Physical education engagement and emotional intelligence played a mediating role in empowering motivational climate in physical education and social adaptation, with a total mediating effect value of 0.251. This study shows that empowering motivational climate in physical education not only directly predicts social adaptation but also indirectly predicts social adaptation through the chain mediating effect of physical education engagement and emotional intelligence.

## Introduction

Social adaptation is the ability of an individual to effectively adapt to the social environment, to be able to independently deal with daily life, and to shoulder social responsibilities (Jingjing et al., 2020). Social adaptation is not only an important index to evaluate individual mental health but also a key factor to determine the degree of individual socialization. It plays an important role in individual academic development, career planning, and future social behavior (Hualing et al., 2021). In December 2016, the National Health Commission of the People’s Republic of China and other government departments issued the “Guiding Opinions on Strengthening Mental Health Services,” stating that: adolescents are the key group of mental health services. Primary and secondary schools should pay attention to students’ mental health education, improve adolescents’ psychological adjustment ability, and maintain good adaptability. Improving students’ social adaptation is also the practical development goal of the school’s physical education curriculum. “General High School Physical Education and Health Curriculum Standards (2017 Edition)” points out that the cultivation of high school students’ health behavior literacy focuses on exercise habits, emotional regulation, and adaptability (Ministry of Education of the People’s Republic of China, 2018). The high school period is a special period in which individual physiology and psychology change rapidly. Due to age characteristics and various reasons, they are also prone to various psychological and behavioral problems in the process of social adaptation (Xinbo et al., 2021). Studies have shown that the development of social adaptation in Chinese adolescents is not ideal, and the proportion of poor social adaptation is higher than the theoretical distribution (Yangang et al., 2008). Based on this, this study intends to focus on the influencing factors and mechanism of social adaptation of high school students, so as to provide a theoretical and empirical basis for promoting their social adaptation.

The social adaptability of adolescents is mainly affected by internal psychological factors (e.g., intelligence level, self-awareness, and personality characteristics) and external objective factors (e.g., school environment, social environment, and family environment), among which the role of school education cannot be ignored. Recent studies have found that school atmosphere (i.e., teacher support, peer support, and autonomy opportunities) is closely related to adolescents’ adaptability, and school atmosphere can directly affect adolescents’ social adaptation (Feilong et al., 2019). Therefore, schools must establish the awareness of cultivating social adaptability, and teachers should create an atmosphere for cultivating social adaptability.

Duda (2013) and Appleton et al. (2016) suggested a hierarchical and multidimensional conceptualization of the coach-created motivational climate that integrates the major social environmental dimensions emphasized within achievement goal theory (AGT) (Nicholls, 1989; Ames, 1992) and self-determination theory (SDT) (Deci and Ryan, 1985, 2000). Duda’s conceptualization suggests that the motivational climate created is multidimensional and can be more or less “empowering” and “disempowering.” The motivational climate created by teachers in physical education has received considerable attention in previous research from an AGT or SDT perspective and holds important pedagogical implications for students’ motivation (Braithwaite et al., 2011), the quality and quantity of their engagement and learning (Reeve, 2012), levels of moderate-to-vigorous physical activity, and psychological responses in physical education (Van den Berghe et al., 2014). The research shows that the empowering motivational climate in physical education is favorable to the development of middle school students’ moral responsibility (Mouratidou et al., 2007). However, the influence of the empowering motivational climate in physical education on high school students’ social adaptation needs to be further confirmed.

### Empowering Motivational Climate in Physical Education and Social Adaptation

The reasons leading to adolescents’ social adaptation disorder may be closely related to the influences of school, family, and society. Among these influencing factors, which factors play a key role in recent years? Some scholars have discussed the relationship between school education and adolescents’ social adaptation, especially focusing on the role of teachers. Previous studies have shown that social adaptation was correlated with specific external factors such as school bonding (Haowen et al., 2021), and the three dimensions of school bonding (i.e., teacher support, peer support, and school belonging) are positively correlated with social adaptation. One study (Ntoumanis, 2001) showed that cultivating intrinsic motivation and providing positive social factors in physical education (e.g., promoting cooperative learning, emphasizing personal progress, and task selection) can produce positive results. Existing studies have shown that compared with other types of support, students who benefit from teachers’ support are more likely to broaden their personal ideological resources and behaviors, which is favorable to adapting to the school environment (Fredrickson and Branigan, 2005). Binrong and Mei (2021) believed that a good teacher–student relationship is conducive to children’s positive emotion toward school, positive behavior, and improvement of individual adaptation. The teacher–student relationship can significantly predict different adaptation types, and the higher the satisfaction of the student–teacher relationship, the less likely it is to become a high-risk or vulnerable type (Yibing, 2018). Therefore, hypothesis 1 is put forward: The empowering motivational climate in physical education can positively predict the social adaptation of senior high school students.

### Mediating Effect of Physical Education Engagement

One of the mediating mechanisms in this study is the mediating effect of physical education engagement. Learning engagement is the superordinate concept of physical education engagement. Learning engagement is a positive cognition and emotional psychological state related to employment and learning. It includes three dimensions, namely, concentration, dedication, and vitality (Schaufeli et al., 2002a). Previous studies have found that school, society, family, and students themselves are the four factors that affect young students’ learning engagement; the school atmosphere has a significant positive correlation with learning engagement (Jingang and Jinze, 2011; Yongzhan, 2016), and teachers’ support has a positive impact on students’ learning engagement (Feng et al., 2017). Research shows that when the teaching environment meets students’ basic psychological needs such as competence, autonomy, and sense of relationship, students’ learning motivation, the sense of well-being, and achievement will be significantly improved, and it also has a promoting effect on students’ learning (Gairns et al., 2015). By creating positive situational stimulation, physical education teachers can improve the students’ motivation level for physical education learning and produce positive results in the improvement of students’ physical health and sports skills, the mastery and application of physical education, and health knowledge, as well as attitude recognition and other learning behaviors (Ntoumanis, 2001). Other studies have confirmed that as a positive psychological index related to students’ learning, physical education engagement reflects the positive psychological state of students in the learning process and plays a very important role in stimulating students’ positive psychological qualities such as social adaptation, optimism, anti-frustration ability, and self-regulation ability (Laitan et al., 2008). Cross-cultural studies show that the more positive adolescents perceive the school atmosphere (i.e., teacher support, peer support, and autonomy opportunities), stronger learning initiative, higher degree of learning engagement, and higher levels of adaptability indicators such as school learning and life satisfaction (Eccles and Roeser, 2011). Therefore, hypothesis 2 is put forward: Physical education engagement plays a mediating role between empowering motivational climate in physical education and senior high school students’ social adaptation.

### The Mediating Effect of Emotional Intelligence

Another mediating mechanism that this study focuses on is the mediating effect of emotional intelligence. Emotional intelligence refers to an individual’s adaptive ability to understand the perceptual utilization and regulation of others’ and their own emotions (Salovey and Mayer, 1990; Schutte and Malouff, 1999). It can also be regarded as both a cognitive ability (Mayer et al., 1999) and a personality trait (Ciarrochi et al., 2001). Studies have shown that school atmosphere (i.e., teacher support, peer support, etc.) has a significant impact on adolescents’ social emotional regulation and problem behaviors (Mingte and Thomas, 2012). There is a significant positive correlation between perceived teacher emotional support and emotional intelligence of high school students (Romano et al., 2020). Other studies have shown that emotional intelligence training programs have a significant impact on college students’ social adaptation (Nimisha and Anoop, 2019). Accurate perception of others’ emotions is associated with good social adaptation, and emotional perception is an antecedent variable of social adjustment. Positive emotion regulation can reduce adolescents’ anxiety level, thus improving individuals’ ability to adapt to the environment (Berking et al., 2008). Studies indicate that adolescent emotional intelligence can not only directly predict social adaptation but also indirectly predict social adaptation through other variables (e.g., shyness, self-esteem, and peer relationship) (Cancan et al., 2011; Qingsong et al., 2016; Zewen et al., 2021). An intervention study shows that while the emotional intelligence of high school students is improved, so is their social adaptation (Lili, 2013; Wenyuan, 2020). Therefore, hypothesis 3 is proposed: Emotional intelligence plays a mediating role between empowering motivational climate in physical education and social adaptation.

### The Chain Mediating Effect of Physical Education Engagement and Emotional Intelligence

As for the physical education engagement and emotional intelligence in this study, some studies have shown that there is a significant positive correlation between high school students’ engagement and emotional intelligence (Hongmei and Lin, 2012; Binghuang and Liang, 2020). Learning engagement is a kind of positive and lasting psychological characteristic of students in the process of learning (Schaufeli et al., 2002b). It reflects the degree of students’ learning engagement and the positive degree of learning psychology. It is conducive to stimulate the positive psychological quality of individuals and promote their development and maturity. Emotional intelligence, which is the ability to properly express, correctly identify, and evaluate emotions, and to regulate one’s own words, actions, and thoughts by using this information, has a significant impact on individuals’ timely self-recognition, positive attitude, and academic learning (Bar-On et al., 2000). Therefore, the two (learning engagement and emotional intelligence) have a collaborative relationship. The support provided by teachers can mobilize students’ learning enthusiasm and improve individual learning engagement, which is conducive to stimulating individual emotional intelligence and improving social adaptation ability. Therefore, hypothesis 4 is proposed: Physical education engagement and emotional intelligence play a chain mediating effect between empowering motivational climate in physical education and social adaptation.

**FIGURE 1:**
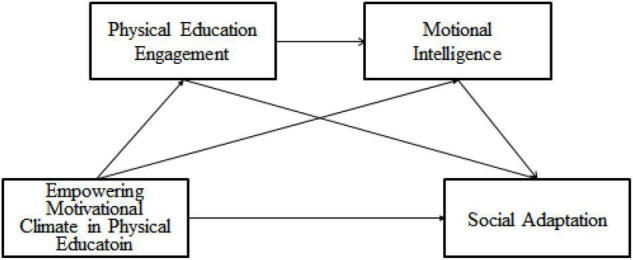
Conceptual framework.

To sum up, this study further deepens and expands previous studies and has theoretical and practical value. On the one hand, this study not only helps to investigate the direct effect of empowering motivational climate in physical education on high school students’ social adaptation but also helps to examine the indirect effect of empowering motivational climate in physical education on high school students’ social adaptation, that is, the independent and chain mediating effects of physical education engagement and emotional intelligence (Figure 1), so as to enrich the research on social adaptation. On the other hand, this study sheds light on why the empowering motivational climate in physical education has an impact on high school students’ social adaptation and how to intervene and cultivate high school students’ social adaptation. It provides a theoretical and empirical basis of psychology for preventing and diagnosing adolescents’ adaptation disorders, improving their social adaptation level, and promoting the better implementation of quality education.

## Materials and Methods

### Participants and Procedure

Using stratified cluster sampling, six high schools were selected from urban and rural areas in the southern, central, and northern Anhui Province, and two classes were randomly selected from each grade of each high school. The questionnaires were distributed to a total of 1,879 students from 36 classes. The main test is for all college students majoring in sports psychology who have received professional training. The consent of the school leaders, teachers, and the test subjects was obtained when the test was administered. The group test was adopted, and the principles of voluntary filling, data confidentiality, and anonymous filling were emphasized, and variables such as gender and grade of the subjects were controlled. Being in accordance with the Declaration of Helsinki, this study has been approved by the Institutional Review Board of the School of Physical Education and Health at Zhaoqing University. In this process, all invited participants are voluntary and confidential.

Notably, 353 invalid questionnaires were excluded due to regular answers, lack of data, and other reasons, and 1,526 questionnaires were considered in this study. The recovery rate was 81.2%. Each questionnaire took about 7–10 min to complete. The average age of the 825 male participants and 701 female participants was 17.00 ± 0.714 years. There were 421 students in the first grade, 589 students in the second grade, and 516 students in the third grade. There was no significant difference in the variables of different genders and grades.

### Measures

#### Empowering Motivational Climate in Physical Education

The empowering motivational climate in physical education was evaluated by the Empowering Motivational Climate Questionnaire in Physical Education (EMCQ-PE), which was a sub-questionnaire of the Empowering and Disempowering Motivational Climate Questionnaire in Physical Education (EDMCQ-PE), and was modified by Pengyu and Jianing (2020) based on Duda’s (2013) EMCQ-PE. The questionnaire has one dimension and 17 questions (e.g., “in PE class, teachers encourage students to cooperate fully in class.”). The Likert’s 7-point evaluation is used, in which 1 represents strongly disagree and 7 represents strongly agree. The higher the score, the higher the level of empowering motivational climate in physical education. A previous study showed that this questionnaire is suitable for high school students (Pengyu and Jianing, 2020). In this study, the internal consistency coefficient α of the questionnaire was 0.89, and the confirmatory factor analysis fit indices were as follows: χ^2^/df = 2.176, SRMR = 0.044, NFI = 0.921, RFI = 0.917, IFI = 0.907, and CFI = 0.929.

#### Physical Education Engagement

The physical education engagement was assessed by the Physical Education Engagement Scale-Student (PEES-S), which was modified by Baogen et al. (2020) based on Laitan et al. (2008) Utrecht Work Engagement Scale-Student (UWES-S). The scale contains a total of 17 items (e. g., “I am full of energy and motivation when learning physical education.”), which is divided into three dimensions: concentration (e.g., “I am immersed in physical education class learning.”), dedication (e.g., “I am passionate about physical education in the classroom.”), and energy (e.g., “I can go on a long time in a physical education class without a break.”). The Likert’s 5-point evaluation is adopted, in which 1 represents very inconsistent and 5 represents very consistent. The higher the score, the higher the level of physical education engagement. Additionally, a previous study demonstrated that this scale was conducted well in a sample of senior high school students (Baogen et al., 2020). In this study, the internal consistency coefficient α of the scale was 0.88, and the confirmatory factor analysis fit indices were as follows: χ^2^/df = 2.245, SRMR = 0.024, NFI = 0.933, RFI = 0.940, IFI = 0.930, and CFI = 0.957.

### Emotional Intelligence

Compiled by Schutte et al. (1998) and revised by Caikang (2002), the Emotional Intelligence Scale (EIS) (Chinese version) was used to measure the emotional intelligence. The research shows that the scale is suitable for the measurement of emotional intelligence of middle school students in China (Caikang and Zhiwen, 2002; Qiuyan et al., 2004). The scale consists of 33 items (e.g., “I can see new hope when I am in a good mood.”), among which questions 5, 28, and 33 are reverse scoring. This scale contains four dimensions, which includes self-emotion regulation (e.g., “When I’m in a good mood, I know how to prolong it.”), emotional perception (e.g., “I find it difficult to read other people’s body language.”), others’ emotional regulation (e.g., “I try to make a good impression on others.”), and emotional use (e.g., “I can control my emotions.”). The Likert’s 5-point evaluation is used, in which 1 means very inconsistent, and 5 means very consistent. The higher the score, the higher the level of emotional intelligence. In this study, the internal consistency coefficient α of the scale was 0.82, and the confirmatory factor analysis fit indices were as follows: χ^2^/df = 2.509, SRMR = 0.031, NFI = 0.915, RFI = 0.904, IFI = 0.927, and CFI = 0.938.

### Social Adaptation

Social adaptation was assessed by the Adolescent Social Adaptation Assessment Questionnaire (ASAAQ), which was compiled by Jianwen and Xiting (2004). The ASAAQ consists of 70 questions (e.g., “I am seen as active”) and contains four dimensions: psychological energy (e.g., “I am a very competitive person.”), psychological dominance (e.g., “I feel I have many strengths.”), psychological resilience (e.g., “I bounce back from bad emotions fairly quickly.”), and interpersonal adaptation (e.g., “I enjoy helping people.”). The Likert’s 5-point evaluation is adopted, in which 1 represents very inconsistent and 5 represents very consistent. The higher the score, the better the social adaptation. The scale has good reliability and validity, and the retest reliability and internal consistency coefficient α are greater than 0.60. Studies have shown that this scale is widely applicable to the measurement of social adaptation of middle school students in China (Xiaohui, 2011; Na, 2015). The internal consistency coefficient α of the scale was 0.86, and the confirmatory factor analysis fit indices were as follows: χ^2^/df = 2.047, SRMR = 0.040, NFI = 0.931, RFI = 0.911, IFI = 0.920, and CFI = 0.940.

### Statistical Analyses

All statistical analyses were performed with IBM SPSS statistical software (version 23.0) and process plug-in by Hayes (2013); confirmatory factor analysis was performed on all questionnaires using Amos 21.0. First, IBM SPSS statistical software version 23.0 was used to test the data for Harman common method deviation; second, Pearson’s correlation analysis was carried out to calculate the relationship among empowering motivational climate in physical education, physical education engagement, emotional intelligence, and social adaptation. Continuous variables of normal distribution are expressed as mean ± standard deviation (SD). Finally, in order to verify the independent and chain mediating effects of physical education learning engagement and emotional intelligence on the relationship between empowering motivation climate in physical education and social adaptation of high school students, model 4 in SPSS macro program compiled by Hayes (2013) was used to test the mediating effect, and model 6 was used to test the chain mediating effect. There is not any missing data in the collected data. There was no significant difference in age and gender of the subjects, so they were not included in the control variable. According to previous experience, the goodness-of-fit index χ^2^/df less than 3, RMSEA less than 0.08, NNFI and CFI greater than 0.9, and SRMR less than 0.05 are acceptable. In this study, the significance level is set as *p* < 0.05.

## Results

### Common Method Deviation Test

Since all of the data in this study were collected from the self-presentation questionnaire survey, and there may be a problem of common method deviation, the questionnaires were filled anonymously, and the purpose of data collection was mainly used for scientific research (Hao and Lirong, 2004). Furthermore, Harman’s single factor method was used to conduct a common method deviation test on the collected data. The results showed that the variance of the first-factor explanation was only 24.53%, which was less than the critical value of 40%. The confirmatory factor analysis method was used to extract a common factor from multiple variables involved in the study, and all items were loaded on this factor. The results showed that the model had a poor data fitting effect: χ^2^/df = 13.67, CFI = 0.51, TLI = 0.46, RMESA = 0.17, SRMR = 0.14, indicating that there is no factor that can explain most of the variation in this study. Therefore, there is no serious problem of common method deviation in this study.

### Descriptive Statistics and Correlation Coefficients for Each Variable

As shown in Table 1, the correlation coefficients of social adaptation, emotional intelligence, physical education engagement, and empowering motivational climate in physical education are all statistically significant. The correlation analysis shows that social adaptation is positively correlated with empowering motivational climate in physical education, emotional intelligence, and physical education engagement.

**TABLE 1:** Average, standard deviation, and correlation coefficient of each variable.

	*M*	SD	1	2	3	4
1. Social adaptation	44.454	6.952	1			
2. Emotional intelligence	4.168	0.615	0.388**	1		
3. Physical education engagement	4.224	0.841	0.295**	0.708**	1	
4. Empowering motivational climate in physical education	6.153	1.074	0.220**	0.577**	0.690**	1

N = 1,526. **p < 0.01.

### Mediating Effect of Physical Education Engagement and Emotional Intelligence

The correlation analysis results met the statistical requirements for further testing the mediating effect of physical education engagement and emotional intelligence (Zhonglin and Baojuan, 2014). Next, the SPSS macro program compiled by Hayes (2013) was made to perform the mediation effect test based on Bootstrap, and the test model 6 was used to conduct the chain mediation model.

**FIGURE 2:**
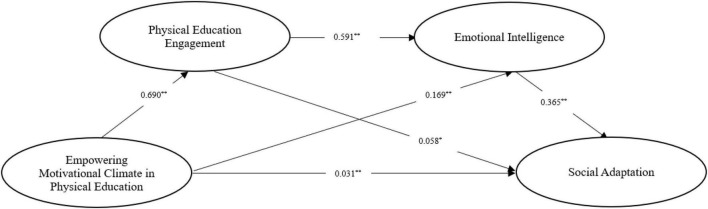
The chain mediation model of empowering motivational climate and school adaptation. *p < 0.05 and **p < 0.01.

Table 2 shows that empowering motivational climate in physical education significantly positively predicts social adaptation (β = 0.282, *p* < 0.01), and hypothesis 1 is established. Next, after incorporating physical education engagement and emotional intelligence into the regression equation, empowering motivational climate in physical education significantly positively predicts the physical education engagement (β = 0.690, *p* < 0.01) and emotional intelligence (β = 0.169, *p* < 0.01). Physical education engagement is significantly positive for emotional intelligence (β = 0.591, *p* < 0.01) and social adaptation (β = 0.058, *p* < 0.05). Emotional intelligence significantly positively predicts social adaptation (β = 0.365, *p* < 0.01). At this time, empowering motivational climate in physical education can still predict social adaptation (β = 0.031, *p* < 0.01). The mediating effect size analysis results show that (see Table 3 and Figure 2) physical education engagement and emotional intelligence are used in empowering motivational climate in physical education and social adaptation. There is a significant mediating effect between adaptation, and the total standardized mediating effect value is 0.251. Mediating effect is specifically composed of indirect effects generated by three pathways: empowering motivational climate → physical education engagement → social indirect effect of adaptation path formation 1 (effect value 0.040); the indirect effect formed by the path of empowering motivational climate → emotional intelligence → social adaptation 2 (effect value 0.062); and empowering motivational climate → physical education engagement → emotional intelligence → social indirect effect 3 of adaptation path (effect value 0.149). The ratios of the three indirect effects to the total effect are 14.18, 21.98, and 52.84%, respectively, and the 95% confidence interval of the above indirect effects does not contain the number 0, indicating that the three indirect effects have reached a significant level, and hypothesis 2, hypothesis 3, and hypothesis 4 are all true. The indirect effect comparison option in model 6 was selected to compare the indirect effects of different paths in pairs to investigate whether there was a significant path difference. Comparison 1 showed that the Bootstrap 95% confidence interval for the difference between indirect effect 1 and indirect effect 2 did not contain 0 value, indicating that indirect effect 1 was significantly different from indirect effect 2. Using the same idea, there is no significant difference between indirect effect 1 and indirect effect 3, indirect effect 2 and indirect effect 3.

**TABLE 2:** Analysis of regression relationship among variables.

Effect	Item	Effect	SE	*t*	*p*	LLCI	ULCI
Direct effect	Empowering motivational climate → social adaptation	0.031	0.040	0.760	0.008	0.011	0.049
Indirect effect process	Empowering motivational climate → physical education engagement	0.690	0.023	30.444	<0.001	0.645	0.734
	Empowering motivational climate → emotional intelligence	0.169	0.030	5.623	<0.001	0.110	0.228
	Physical education engagement → emotional intelligence	0.591	0.030	19.639	<0.001	0.532	0.650
	Physical education engagement → social adaptation	0.058	0.047	1.230	0.019	0.034	0.149
	Emotional intelligence → social adaptation	0.365	0.041	8.817	<0.001	0.284	0.447
Total effect	Empowering motivational climate → social adaptation	0.282	0.031	9.097	<0.001	0.160	0.280

*All variables in the model have been standardized, the same as below; LLCI is the lower limit of 95% interval of estimated value, and ULCI is the upper limit of 95% interval of estimated value.*

**TABLE 3:** Mediating effect analysis of empowering motivational climate and school adaptation.

Item	Effect	Boot SE	Boot LLCI	Boot ULCI	*p*
Empowering motivational climate → physical education engagement → social adaptation	0.040	0.003	0.068	0.077	<0.001
Empowering motivational climate → emotional intelligence → social adaptation	0.062	0.014	0.037	0.09	<0.001
Empowering motivational climate → physical education engagement → emotional intelligence → social adaptation	0.149	0.010	0.132	0.172	<0.001
					

## Discussion

### Empowering Motivational Climate and Social Adaptation

This study focuses on the mechanism of empowering motivational climate in physical education on social adaptation of high school students. The results show that empowering motivational climate in physical education significantly positively predicts school adaptation, and the independent and chain mediating effects of physical education engagement and emotional intelligence are verified. This study verifies that empowering motivational climate in physical education is a significantly positive predictor of social adaptation, which is consistent with previous studies (Lim and Wang, 2009), and verified hypothesis 1. Physical education teachers can build a positive psychological environment to meet the basic needs of students and further produce adaptive behavior results through physical exercise motivation (Lim and Wang, 2009). Research by Jose et al. (2020) shows that teachers are more closely related to school adaptation among the social support perceived by adolescent students from family, teachers, and friends. Other studies have shown that teacher caring behavior can significantly positively predict the social adaptation of ethnic minority students (Yaqiong et al., 2019). There is a significant positive correlation between social support (teachers, classmates, and relatives) and social adaptation, and the reduction of adolescents’ perceived social support will lead to a decrease in their social adaptation (Yan et al., 2017). One possible reason is that students who can positively perceive emotional support from teachers are better able to resist adjustment barriers and school burnout than other students (Reschly et al., 2008). Other studies have confirmed that positive social factors provided by physical education teachers (e.g., teachers encouraging students to try new sports skills and encouraging students to cooperate fully in sports) can satisfy students’ sense of relationship, competence and autonomy and then produce more positive behaviors through motivation (Deci and Ryan, 1985, 2000; Lim and Wang, 2009). Therefore, empowering motivational climate in physical education plays an important role in promoting high school student association adaptation. In physical education class, teachers’ care and encouragement, appreciation, and trust, as well as various teaching organizations and teaching methods, may promote the cultivation of high school students’ social adaptation ability.

### Independent Mediating Effect of Physical Education Engagement and Emotional Intelligence

This study not only investigates the direct relationship between empowering motivational climate in physical education and high school students’ social adaptation but also builds a mediation model between them using physical education engagement and emotional intelligence as the mediating variables. It is found that physical education engagement plays a partial mediating role between empowering motivational climate and social adaptation. Hypothesis 2 is verified. This is related to the previous research evidence about which empowering motivational climate has a positive effect on physical education engagement (Reeve, 2012) which contributes to social adaptation (Tao and Zheng, 2006; Wenhua et al., 2013). This study investigates the relationship among the above three variables simultaneously, revealing that empowering motivational climate in physical education is an important factor to improve the learning input of physical education. It is also an important factor for social adaptation. The possible reason is that senior high school students who perceive sufficient support from physical education teachers show a high level of physical education engagement and enhance the communication among classmates in the process of high-quality physical education learning. The psychological compatibility among students is improved, and the self-confidence and anti-frustration ability are enhanced. The psychological quality of students is improved, so is their social adaptation. It is worth mentioning that, as a positive emotional state, physical education engagement enables individuals to correctly deal with the challenges they encounter in the learning process. This is not only conducive to the improvement of high school students’ optimism and self-esteem but also helpful to buffer external pressure. In other words, physical education engagement can enable individuals to maintain a good physical and mental state. Individuals with physical education engagement have higher levels of physical and mental health (Steele and Fullagar, 2009).

It is also found that emotional intelligence plays a partial mediating role between empowering motivational climate in physical education and social adaptation, which was consistent with existing research results and verified hypothesis 3. Empowering motivational climate in physical education plays an important role in promoting emotional intelligence (Sergio et al., 2020). The possible reason is that perceived teacher emotional support by high school students is more closely associated with lower levels of emotional exhaustion and higher subjective well-being than other sources of support (Hughes et al., 2001, 2008). Studies have shown that emotional intelligence can positively predict social adaptation (Engelberg and Sjöberg, 2004; Jiajun et al., 2016); individuals with high emotional intelligence are better able to adjust their emotions, perceive their own and others’ emotions more accurately, better cope with the pressure and confusion brought by adverse environments, keep an optimistic attitude in difficult situations, and better adapt to the society (Yu and Peng, 2017). The results of this study confirm that the motivation climate of PE empowerment is an important factor in promoting not only emotional intelligence but also social adaptation of high school students. According to the stress coping model, high school students with sufficient support from teachers can acquire emotional management ability related to emotional disclosure, which will help them regulate anxiety, depression, and other negative emotions more effectively, and then improve social adaptation. In terms of the influence of emotional intelligence on individual life, the important role of emotional intelligence is characterized by two aspects: on the one hand, emotional intelligence should be embodied in the areas of emotional activities, such as dealing with setbacks and challenges encountered in our daily life, and on the other hand, emotional intelligence should deal with things and individual efficiency and the ability to combine.

### Chain Mediating Effect of Physical Education Engagement and Emotional Intelligence

This study further found that physical education learning engagement and emotional intelligence had a chain-mediated effect on the relationship between empowering motivational climate and social adaptation, which verified hypothesis 4. This is consistent with the existing research results that physical education engagement contributes to emotional intelligence (Hongmei and Lin, 2012). Previous studies have shown that emotional intelligence and physical education engagement have a significant positive correlation, but most studies have confirmed that emotional intelligence can significantly and positively predict physical education engagement (Binghuang and Liang, 2020). This study found that physical education engagement also significantly positively predicted emotional intelligence. Learning engagement is a continuous, full, positive, and perfect mood and state of employees, which is characterized by vitality, dedication, and concentration (Schaufeli et al., 2002a). The higher the degree of learning involvement, the more persistent efforts in the face of difficulties, full of enthusiasm for learning, and dare to face the challenges in learning. Such positive and healthy emotions and cognition can help individuals to timely understand themselves, maintain a positive attitude, deal with various pressures and difficulties in the environment, monitor their own and others’ emotions, and then promote the development of their own emotional intelligence. Through the chain mediation effect test, this study found that adequate physical education teacher support can promote students to fully engage in learning, which will lead to a healthier development of emotional intelligence, and then promote social adaptation. Therefore, in physical education teaching, teachers should create a good learning environment through a variety of teaching methods or teaching modes, improve the level of the students’ physical education engagement, which in turn raises the student good emotional intelligence, in order to better cope with problems and challenges brought about by the pressure, maintains an optimistic attitude in the difficult situation, and finally improve the social adaptability of high school students.

Empowering motivation climate can stimulate senior high school students’ learning motivation and then promote physical education engagement, and physical education learning engagement and emotional intelligence are significantly positive correlation. Therefore, the chain mediation of physical education engagement → emotional intelligence in this study is feasible and has a partial mediating effect between the empowering motivation climate and social adaptation. To a certain extent, the mediating effect model reveals the mechanisms underlying the association between empowering motivational climate and social adaptation. It is suggested that on the premise of giving full play to the role of empowering motivation climate, physical education educators can further improve the social adaptability of senior high school students by improving their physical education investment and emotional intelligence.

### Practical Significance

First, the empowering motivational climate in physical education is an important variable to predict social adaptation. Adolescence is a critical period for a person’s development, and it is the period with the most changes in the life cycle. It keeps growing in physical and psychological aspects, and the individual’s mentality and social relations are constantly changing. Due to age characteristics and other reasons, they are prone to adaptive disorders and problematic behaviors. School education is an effective process to accelerate, guide, and standardize individual socialization, and the cultivation of adolescents’ good social adaptability should be an important part of school education. Teachers have to shoulder the heavy responsibility of not only spreading cultural skills but also instilling new social role norms, and also creating an atmosphere of social adaptation, and teaching students to adjust themselves with social rules. Second, physical education engagement and emotional intelligence are the important factors that affect the social adaptation of high school students. Schools should take practical and effective measures to cultivate emotional intelligence. On the one hand, schools should establish a new teacher–student relationship, build an emotional classroom teaching model, set emotional intelligence education content in standardized courses, and adopt a scientific teaching evaluation system. On the other hand, they should carry out humanities education, social practice activities, quality development training, and professional school psychological consultation. In addition, schools should also organize a variety of extracurricular sports activities and sports competitions. Teachers can use a variety of teaching content, teaching methods, teaching organization, and teaching evaluation to improve the endogenous motivation of physical education courses and then increase students’ level of physical education engagement.

### Limitations and Future Directions

This study explores the relationship between empowering motivational climate in physical education and senior high school students’ social adaptation and constructs a chain mediation model. It reveals the mechanisms underlying the association between empowering motivational climate and social adaptation. It has important theoretical and practical value to understand the causes of senior high school students’ social adaptation, and at the same time, it also provides a basis for studying the causal relationship between the variables. However, this study has not been able to draw a causal inference between the variables. Longitudinal tracking or experimental intervention design can be used in the future, so as to more effectively explain the impact of empowering motivational climate in physical education on senior high school students’ social adaptation. In addition, in this study only physical education engagement and emotional intelligence are considered in empowering motivational climate. In fact, there may still be other intermediary variables such as personality, self-esteem, and so on, which need to be further explored in the future. In the future research, we will adhere to the education concept of “health first” and explore the multiple mechanisms of physical education on adolescent physical and mental health.

## Conclusion

Empowering motivational climate in physical education can significantly positively predict social adaptation. Physical education engagement and emotional intelligence play a significant mediating role between empowering motivational climate in physical education and social adaptation. There are three mediating paths, namely, the separate mediating effect of physical education engagement, the separate mediating effect of emotional intelligence, and the chain mediating effect of physical education engagement and emotional intelligence.

## Data Availability Statement

The original contributions presented in the study are included in the article/Supplementary Material, further inquiries can be directed to the corresponding authors.

## Author Contributions

KG designed the study, collected and analyzed the data, and wrote the manuscript. QM, SY, HC, ZH, CL, and PW revised the manuscript. All authors contributed to the article and approved the submitted version.

## Conflict of Interest

The authors declare that the research was conducted in the absence of any commercial or financial relationships that could be construed as a potential conflict of interest.

## Publisher’s Note

All claims expressed in this article are solely those of the authors and do not necessarily represent those of their affiliated organizations, or those of the publisher, the editors and the reviewers. Any product that may be evaluated in this article, or claim that may be made by its manufacturer, is not guaranteed or endorsed by the publisher.
